# Functionalized-Graphene Field Effect Transistor-Based Biosensor for Ultrasensitive and Label-Free Detection of β-Galactosidase Produced by *Escherichia coli*

**DOI:** 10.3390/bios13100925

**Published:** 2023-10-12

**Authors:** Shanhong Wei, Yanzhi Dou, Shiping Song, Tie Li

**Affiliations:** 1 State Key Laboratory of Transducer Technology, Shanghai Institute of Microsystem and Information Technology, Chinese Academy of Sciences, Shanghai 200050, China; weish@mail.sim.ac.cn (S.W.); douyanzhi@mail.sim.ac.cn (Y.D.); 2 University of Chinese Academy of Sciences, Beijing 100049, China; 3 Shanghai Institute of Applied Physics, Chinese Academy of Sciences, Shanghai 201800, China; 4 Institute of Materiobiology, College of Science, Shanghai University, Shanghai 200444, China

**Keywords:** GFET, β-galactosidase detection, ultrasensitive, label-free

## Abstract

The detection of β-galactosidase (β-gal) activity produced by *Escherichia coli* (*E. coli*) can quickly analyze the pollution degree of seawater bodies in bathing and fishing grounds to avoid large-scale outbreaks of water pollution. Here, a functionalized biosensor based on graphene-based field effect transistor (GFET) modified with heat-denatured casein was developed for the ultrasensitive and label-free detection of the β-gal produced by *E. coli* in real water samples. The heat-denatured casein coated on the graphene surface, as a probe linker and blocker, plays an important role in fabricating GEFT biosensor. The GFET biosensor response to the β-gal produced by *E. coli* has a wide concentration dynamic range spanning nine orders of magnitude, in a concentration range of 1 fg·mL^−1^–100 ng·mL^−1^, with a limit of detection (LOD) 0.187 fg·mL^−1^ (1.61 aM). In addition to its attomole sensitivity, the GFET biosensor selectively recognized the β-gal in the water sample and showed good selectivity. Importantly, the detection process of the β-gal produced by *E. coli* can be completed by a straightforward one-step specific immune recognition reaction. These results demonstrated the usefulness of the approach, meeting environmental monitoring requirements for future use.

## 1. Introduction

Pathogenic microorganisms include bacteria, viruses, fungi, etc., which can cause various diseases. This is especially the case for bacteria, such as *E. coli*, *Salmonella,* and *Vibrio cholerae,* present in seawater bodies in bathing and fishing grounds; these can cause environmental water pollution and are a threat to public health and safety [1,2,3]. The rapid and sensitive detection of *E. coli* in water samples is critical for ensuring that water sources are safe, minimizing the risk of human exposure to potential hazards. Nowadays, *E. coli* is one of the most widely studied bacteria; it can be used as a potential marker for the presence of pathogens originating from environmental water pollution [4,5]. In particular, β-galactosidase (β-gal), encoded by the lacZ gene of the *E. coli* chromosome and composed by four noncovalently bound subunits of 116 kDa each, together with the capacity of the organism to metabolize lactose, is important for the heterotrophic growth of *E. coli*. In the presence of lactose, β-gal is produced by *E. coli* to hydrolyze lactose into galactose and glucose, which is then used as a carbon source. Compared with direct detection of *E. coli*, the sample acquisition and processing of galactosidase is simpler and faster. β-gal has been widely utilized to monitor the concentration of *E. coli* as a metric for the safety of water sources [6,7,8]. Therefore, a fast and sensitive β-gal detection method is urgently needed to monitor whether water sources are contaminated in the field environment.

Currently, various sensitive analytical methods, including electrochemistry, bioluminescence, dynamic light scattering (DLS), and surface plasmon resonance (SPR) are available for β-gal and *E. coli* quantitative detection in different samples [6,9,10,11,12,13,14,15,16,17,18]. Olivier Laczka et al. have proposed the efficiency of methodologies for the detection of enteric pathogens, based on the amperometric detection of β-gal activity. A measure of 10 CFU·mL^−1^ can be detected in river water samples. Better limits of detection were achieved with this strategy in the absence of phages. This is a simple one-step method for detecting fecal contamination in water sources, with less user manipulation of the sample, but it still takes one day to complete the test. Thomas Thund et al. have developed the detection of *E. coli* in orange juice using a portable nanofiber light-addressable potentiometric sensor. The successful detection of *E. coli* was reported with the nanofiber light-addressable potentiometric sensor in less than 1 h. The LOD measured in the sensor was found to be 10^2^ CFU/mL. The biosensor has good selectivity for the detection of *E. coli.* Sean Burnham et al. have proposed a strategy: wild-type T4 bacteriophage and recombinant reporter lac Z T4 bacteriophage carrying the β-gal gene can be used for the detection of generic *E. coli* by monitoring the release of β-gal upon phage-mediated cell lysis. The *E. coli* concentration as low as 40 or <10 CFU·mL^−1^ can be detected visually within 8 h, respectively. Application of the bioluminescent β-gal substrate allowed reliable detection of <10 CFU·mL^−1^ within 5.5 h. Shengnan Zhan et al. described for the first time an improved no-wash immunosensor based on dynamic light scattering for the detection of *E. coli O157:H7* in milk using GNFs for sensitive signal transduction, with a limit of detection of 2.7 CFU/mL. The GNF-DLS immunosensor constructed by the authors can be used for rapid and highly sensitive detection of pathogenic bacteria; this can be extended to ultrasensitive detection of other trace analytes. Allen D. Taylor et al. have reported on the quantitative and simultaneous detection of four species of bacteria—*E. coli O157:H7*, *Salmonella choleraesuis serotype typhimurium*, *Listeria monocytogenes*, and *Campylobacter jejuni*—using an eight-channel surface plasmon resonance (SPR) sensor based on wavelength division multiplexing. The LODs for each of the four species of bacteria ranged from 3.4 × 10^3^ to 1.2 × 10^5^ CFU·mL^−1^. Even though the employed methods above have the benefits of high sensitivity and selectivity, they suffer from some disadvantages, like lengthy procedures, use of sophisticated instruments, and the requirement for trained personnel [4,19,20]. Therefore, researchers are constantly investing a lot of effort in developing new analytical methods to improve the analytical performance of bacterial surveillance.

Electromechanical FET biosensors, fabricated by micro- and nano-processing technologies, such as silicon nanowire (SiNW) FET, multi-walled carbon nanotubes (MWNTs) FET, and graphene FET (GFET), can be used for the detection of various biological targets; this has many inherent advantages over other sensing technologies, including easy integration and mass production, high sensitivity, and label-free detection [21,22,23,24,25,26,27]. The small alteration in the parameter induces a noticeable change of channel current due to combining efficient transducers with signal amplifiers [28,29]. Graphene is a two-dimensional honeycomb lattice of carbon with unique electronic, physical, and chemical properties for biosensing compared to other nanomaterials, such as high intrinsic carrier mobility, ambipolar effect, low intrinsic noise level, good biocompatibility, and stability, and it has been employed in fabricating FET devices [30,31]. Liqian Wang et al. reported a graphene FET molecular system consisting of an aptamer probe bound to a flexible single-stranded DNA cantilever linked to a self-assembled stiff tetrahedral double-stranded DNA structure for the rapid and ultrasensitive electromechanical detection of unamplified nucleic acids, ions, small molecules, and proteins in biofluids. Electromechanical biosensors are integrated and portable prototype devices; a study has shown that such a device detected SARS-CoV-2 RNA without the need for RNA extraction or nucleic acid amplification [32]. Shun Wang et al. developed an ultrasensitive antibiotic detection method based on an aptamer-functionalized ultraclean graphene FET biosensor. A dynamic detection range of 5 orders of magnitude, a sensitivity of 21.7 mV/decade, and a low detection limit of 100 fM were achieved for GFET biosensors with good stability [33]. Shicai Xu et al. showed a single-crystal domain patterned into a multi-channel graphene biosensor for real-time reliable determination of binding kinetics of DNA hybridization with the detection limit of 10 pM. It can distinguish single-base mutations quantitatively in real time. They have developed an analytical model to estimate probe density, efficiency of hybridization, and the maximum sensor response. The GFET biosensor array can be used for cost-effective, high-throughput screening of disease biomarkers, equipping it with a wide range of application prospects in the field of biology and medicine [34]. These studies show that graphene FET biosensors functionalized with biological molecules and nanomaterials have excellent sensing performances for detecting various targets.

Herein, a functionalized GFET electromechanical biosensor modified with heat-denatured casein is developed for ultrasensitive and label-free detection of β-gal produced by *E. coli*. The GFET has the high selectivity to β-gal, and the ultra-sensitivity which is 3 orders of magnitude below the detection limit of current optical detection methods. The GFET displays excellent sensing performance for the detection of β-gal due to the intrinsic characteristic of single-layer graphene, such as high electron mobility and π–π stacking interaction with biological molecules, and the good antifouling ability of heat-denatured casein. Moreover, casein modified on the surface of graphene, as a molecular linker, can construct a universal sensing interface to link different biological probes and realize the detection of a variety of target molecules through specific recognition reactions, which has broad application prospects in the field of sensing.

## 2. Materials and Methods

### 2.1. Materials

Casein, phosphate-buffered saline (PBS, pH 7.4), N-hydroxysuccinimide (NHS), 1-ethyl-3-(3-dimethylaminopropyl)-carbodiimide (EDC), horseradish peroxidase (HRP), and glucose oxidase (Gox) were purchased from Sigma-Aldrich, (St. Louis, MO, USA). The single-layered graphene film grown on copper film using chemical vapor deposition process were obtained from Vigon material (Hefei, Anhui, China). The protein of β-gal and anti-β-gal antibody from *E. Coli* were purchased from Abcam (Cambridge, UK). Casein was dissolved in 10 nM PBS and all solutions were prepared in deionized water. Water sample collected from river in Xuhui District Shanghai was used after removing interference from solid impurities by standing for 1 h in the laboratory.

### 2.2. Fabrication of Graphene Device

The silicon wafer was employed as the substrate of the GFET, and then a dry oxygen oxidation was performed to form a 300 nm thick silica layer upon silicon wafer. The source and drain electrodes consisting of a Ti/Au structure (10 nm/100 nm) were defined on the silica surface by photolithography, metal deposition, and a lift-off process. The copper film underneath the graphene film was corroded by aqueous ferric chloride. After washing with deionized water, the graphene film with poly (methyl methacrylate) (PMMA) on the top was transferred onto the wafer surface with Ti/Au pattern. The PMMA layer was eliminated by acetone solution. Followed by photolithography and O_2_ plasma process, the graphene channel was formed. After removing the photoresist which served as the graphene mask layer, the wafer with graphene channels was annealed under the atmosphere of H_2_ and Ar at 300 °C for 1 h. Finally, the wafer was sliced, and the device was packed by silica gel and silicone tube.

### 2.3. Functionalization of Graphene Surface

To functionalize the graphene surface, the GFET was first immobilized with heat-denatured casein. The GFET was immersed in 10 μg·mL^−1^ casein solution for 3 min under 90 °C. Then, the device was rinsed with PBS to remove any free casein. The heat-denatured casein works as the linker containing carboxylic acid groups and can irreversibly interact with graphene by pi-stacking. Followed by the incubation of a mixture of 2 mg·mL^−1^ anti-β-gal antibody solution, 5 mg·mL^−1^ EDC, and 1 mg·mL^−1^ NHS overnight at room temperature in the dark, the device was rinsed with PBS. To block the excess reactive groups, 10 mg·mL^−1^ casein solution was added onto the graphene surface for 1 h at room temperature. Finally, the device was rinsed with deionized water and dried in N_2_ atmosphere for subsequent experimentation.

### 2.4. Characterization and Measurement

A semiconductor parameter analyzer (Keithley 4200, Keithley, Solon, OH, USA) was employed to apply the electric field. With an aqueous solution-gate applied, the source–drain current (I_ds_) was measured under the bias of drain–source voltage (V_ds_, 0.1 V) and gate–source voltage (V_gs_, −0.1 V). During the electric measurements process, an Ag/AgCl reference electrode served as the gate electrode was inserted in the aqueous solution at a fixed position. To compare GFETs, the change value of I_ds_ was normalized by ΔI/I_0_ = (I_1_ − I_0_)/I_0_, where I_0_ is the current value of blank sample and I_1_ is the current value of corresponding sample. The surface topology of graphene film and the thickness of heat-denatured casein film were acquired with atomic force microscopy (AFM) (Bruker, Hamburg, Germany). Raman spectra was conducted by Bruker (Hamburg, Germany). The wettability of graphene was obtained using a contact angle measuring system (Dataphysics, Filderstadt, Germany).

## 3. Results and Discussion

### 3.1. Verification of GFET

**Figure 1:**
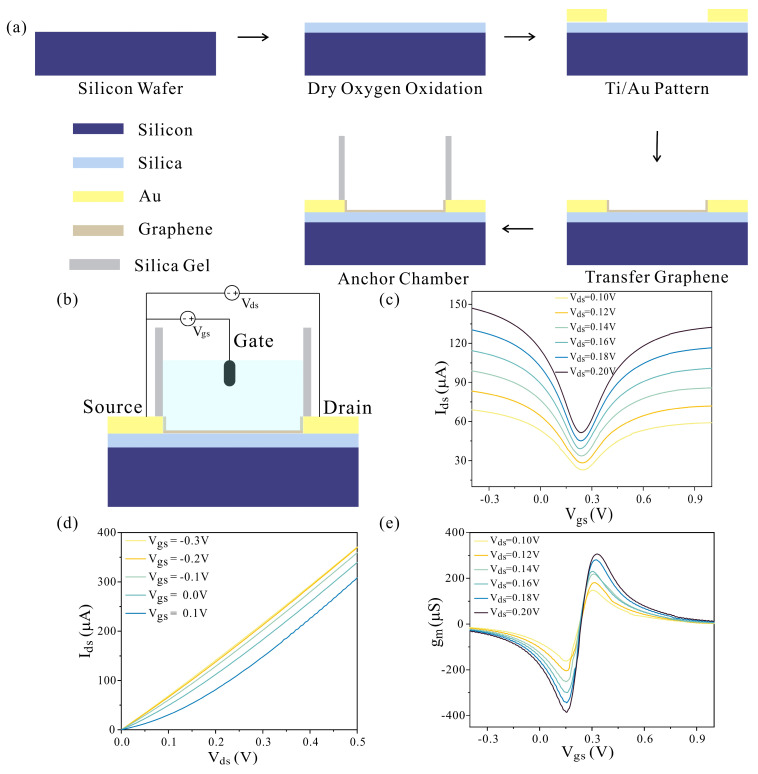
The fabrication and electrical characterization of the GEFT. (**a**) The fabrication protocol of the GFET. (**b**) The GFET device with source, drain, and gate was used in this study. The gate voltage was applied through Ag/AgCl reference electrode which was immersed in the solution. The source voltage and drain voltage were applied by the contact pads. (**c**) Transfer characteristics of GFET. (**d**) Output characteristics of GFET. (**e**) GFET at different V_ds_ was defined as the derivative of I_ds_ with respect to V_gs_.

The detail-fabrication processes of the graphene devices are provided in Figure 1a. In the process of fabrication of graphene device, followed by a dry oxygen oxidation, Ti/Au were patterned on the surface of wafer as source and drain electrodes by photolithography, metal deposition, and a lift-off process. Then, the graphene film peeled from copper film was transferred onto the wafer surface to serve as the channel of device. After removing the PMMA film on graphene film by acetone solution, the graphene was etched by O_2_ plasma to pattern the channel. The wafer was sliced after annealing step. Finally, the graphene device was packed using silica gel for subsequent research on sensing applications. The sensing region dimensions of the fabricated graphene device were 5 × 5 μm^2^ (L × W). To verify the ambipolar field effect semiconductor-like behavior of aqueous-solution-gated GFET (Figure 1b), the transfer characteristics and the output characteristics were measured. As shown in the transfer characteristic curve (Figure 1c), the current value increased with the increase in V_gs_ on both sides of Dirac point which is consistent with previous studies [35,36]. Additionally, the Dirac point voltage was around 0.25 V. In the output characteristic curve (Figure 1d), the I_ds_ increased with the increase in V_ds_ at different V_gs_ from −0.3 V to 0.1 V, indicating the successful transfer of graphene and the construction of device. The transconductance (g_m_) positively correlated with the device sensitivity or responsivity is a key parameter for GFET [37]. The g_m_, defined as the derivative of I_ds_ with respect to V_gs_, is recorded in Figure 1e. At V_ds_ = 0.2 V, the g_m_ of GFET reached a negative maximum value of −386.2 μS in the hole regime and a positive maximum value of 306.1 μS in the electron regime, indicating the gate voltage in the hole regime could be a better choice for target detection. Further, the hole mobility and electron mobility of GFET were estimated to be −10,388.6 and 8232.6 cm^2^·V^−1^·s^−1^, respectively, which is way better than that of other devices [35,38].

### 3.2. Characterization of Functionalized GFET

**Figure 2:**
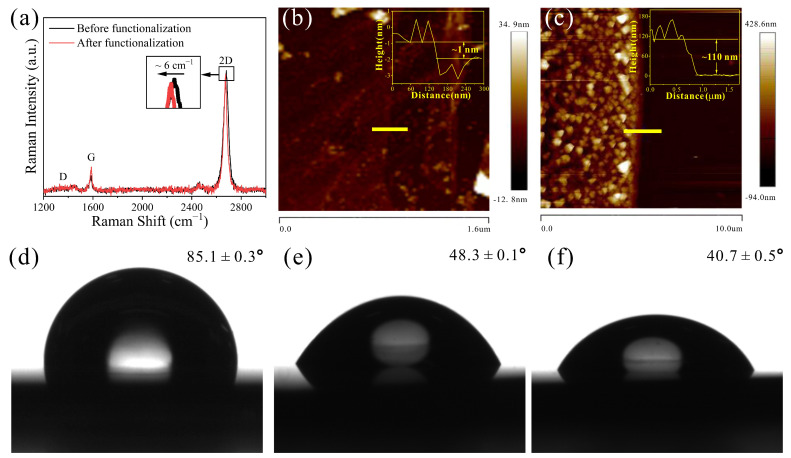
(**a**) Surface analysis of pristine and heat-denatured casein-modified graphene using Raman spectra. (**b**) AFM image of pristine graphene. Inset shows that the thickness of pristine graphene. (**c**) AFM image of graphene modified with heat-denatured casein. Inset shows that the thickness of graphene modified with heat-denatured casein. The contact angle image of (**d**) pristine, (**e**) heat-denatured casein-modified, and (**f**) anti-β-gal-modified graphene.

The functionalization of the GFET was prepared with casein solution through heat denaturing at 90 °C for 3 min. To confirm that the morphology, composition, and size of the graphene surface was functionalized using casein solution, we obtained Raman spectra, AFM, and contact angle test dates (Figure 2a–c). Figure 2a showed the characteristic D, G, and 2D bands of graphene, i.e., 1353.21 cm^−1^, 1587.98 cm^−1^ and 1884.13 cm^−1^, respectively. The ratio of the intensity 2D band and G band (𝐼_2D_/𝐼_G_) was 4.76 in the pristine graphene, and the spectrum was consistent with other monolayer graphene spectra, which indicates that the graphene film was monolayer [39,40]. Raman spectroscopic analysis of the graphene surface after the modification of heat-denatured casein indicated that the doping of the graphene was n-type [41]. Before performing the antibody modification, graphene surface topography characterization was conducted using AFM to verify the successful functionalization of heat-denatured casein. As shown in Figure 2b,c, the thickness of the graphene was found to increase from 1 nm to 110 nm with treatment of heat-denatured casein; from this, it can be concluded that the heat-denatured casein was successfully immobilized on graphene surface. The wettability of the graphene can be changed by the modification of protein, which can verify the graphene surface of the functionalization process. Figure 2d–f present the results of the contact angle measurement from the pristine graphene, the heat-denatured casein-modified graphene, and the anti-β-gal-antibody-modified graphene, respectively. The pristine graphene exhibited a high contact angle of 85.1 ± 0.3°, demonstrating the highly hydrophobicity of the graphene. The contact angle of the heat-denatured casein-modified graphene decreased to 48.3 ± 0.1° immediately, indicating the reliable adhesion of the heat-denatured casein on the graphene surface. When the anti-β-gal antibody was covalently modified on the graphene surface through chemical coupling, the contact angle was 40.7 ± 0.5°, which was a slight decrease in the contact angle, suggesting the successful modification of anti-β-gal antibody on the graphene’s surface.

### 3.3. Sensing Capability of GFET to Detect β-Gal Produced by E. coli

**Figure 3:**
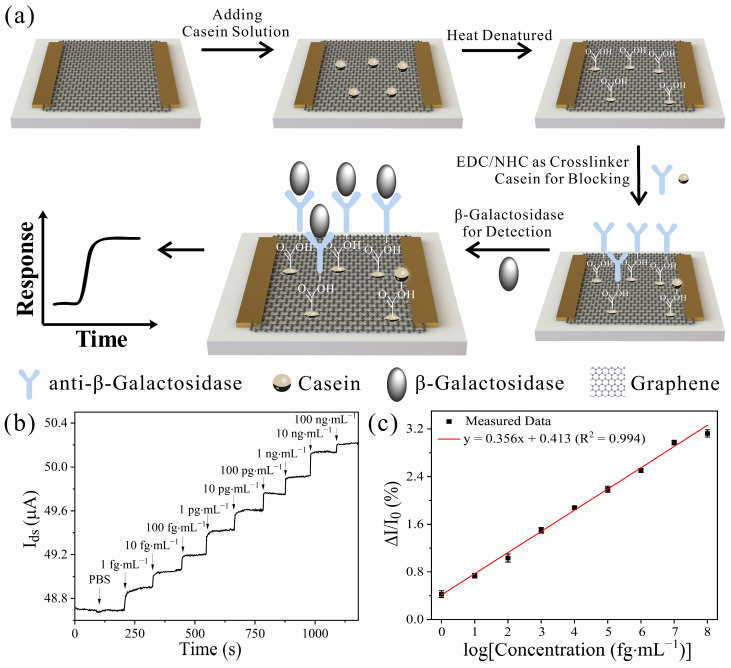
Detection of β-gal produced by *E. coli*. (**a**) Schematic illustration of β-gal detection with GFET. Graphene served as the sensing material in the GFET device, and anti-β-gal is modified on the graphene surface through heat-denatured casein film as a probe linker. (**b**) Real-time response of the GFET toward β-gal in the concentration range from 1 fg·mL^−1^ to 100 ng·mL^−1^ in PBS solution and (**c**) related dose-dependent response curve. Graphene-based FET that did not detect β-gal is presented as negative control (V_ds_ = 0.1 V). Error bars represent the standard deviation of three replicates.

A schematic illustration of the fabrication of the functionalized GFET biosensors for the β-gal detection is shown in Figure 3a. The graphene served as a sensing material in the GFET device, and the anti-β-gal antibody was modified on the graphene surface through heat-denatured casein film. In order to fix the anti-β-gal antibody onto the functionalized graphene surface, we first used heat-denatured casein as a probe linker to generate more carboxyl groups on the graphene surface, and activated the carboxyl groups through EDC/NHS [42]. The channel conduction of the GFET changed because charged β-gal is specifically captured by the anti-β-gal antibody on the graphene surface, resulting in a corresponding change in the source–drain current. Sensitivity for β-gal produced by *E. coli* detection determines the sensing capability of the GFET biosensors. Utilizing the optimal conditions in our previous work, the detection ability of GFET in the presence of different concentrations of β-gal was investigated [43]. As displayed in Figure 3b, a clear change of current from blank sample to different concentration of β-gal could be obviously differentiated; that is, the current value was increased along with the β-gal concentration. It could be that β-gal—specifically bound to the anti-β-gal antibody on the graphene surface—was negatively charged in the PBS solution, leading to the holes being induced in the graphene. Due to the fact that the main carriers in the graphene were the holes when Vgs was biased at −0.1 V, I_ds_ increased along with the accumulation of the holes. As shown in Figure 3c, ΔI/I_0_ was linearly related to β-gal in the logarithm of the concentration range from 1 fg·mL^−1^ to 100 ng·mL^−1^, which spans 9 orders of magnitude. The linear regression equation of β-gal can be described as y = 0.356x + 0.413 with a correlation of 0.994 (R^2^ = 0.994), where y is ΔI/I_0_, and x is the base-10 logarithm of the concentration of β-gal. Furthermore, the detection limit was found to be 0.187 fg·mL^−1^ [27]. That is to say that we could detect the β-gal concentration of 1.61 aM, which is ultrasensitive to the detection of environmental water samples. The detection ability of the GFET biosensor to β-gal is close to the single-molecule scale. The ultra-sensitivity of GFET may be attributable to the synergistic effect of the high sensitivity of graphene to its surface change and the distinguished pollutant-resistant nature of heat-denatured casein.

### 3.4. Specificity of GFET and Real Sample Analysis

**Figure 4:**
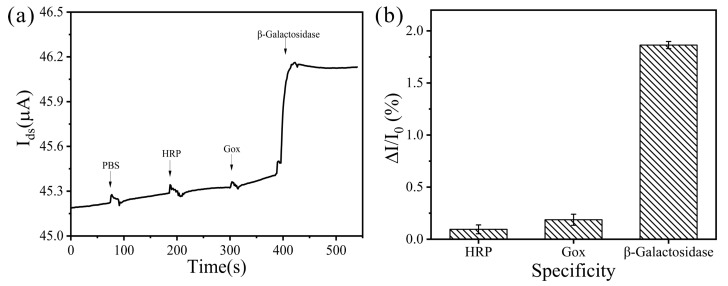
(**a**) The specific response of β-gal based on GFET biosensor, compared to the respective signal responses of the targets (HRP, Gox, and β-gal) at the same concentration levels (10^4^ fg·mL^−1^). The PBS solution represents the negative control; (**b**) related response bar graph. Error bars represent the standard deviation of three replicates.

The specificity of the GFET biosensor chip in detecting β-gal is also an important parameter for the performance of the biosensor. Good specificity is helpful in improving the accuracy of the target molecules detected by biosensors. To investigate the specificity of the GFET biosensor, two proteins were used as interference proteins to examine the cross-reaction: HRP and Gox. The PBS solution was used as a blank reagent. Employing the developed GFET with β-gal, we recorded their current responses (Figure 4). In theory, none of these proteins initiate the binding reaction with anti-β-gal; thus, no I_ds_ change occurs. As shown in Figure 4a, the change of I_ds_ in interference proteins was similar to that in the PBS sample, and the value of I_ds_ in β-gal is considerably larger than that of other proteins, as expected. The results indicate the high specificity of GFET toward β-gal and they show that the GFET can distinguish β-gal from other proteins due to the specific recognition of the anti-β-gal antibody for β-gal; additionally, the heat-denatured casein film served as a protective layer on the graphene film.

River water is a kind of sample that can be conveniently obtained and tested for the detection of various pathogens in contaminated water. The practical performance of the GFET was validated with a real river water sample. The accuracy of the developed GFET was evaluated by determining the recoveries of β-gal in the river water samples by the standard addition method and analyzing each sample with three independent experiments. The river water samples were spiked with β-gal at concentrations of 1, 10, and 100 ng·mL^−1^. The spiked samples were detected by the GFET. As can be seen in Table 1, the recoveries of β-gal in the river water samples were between 92.710% and 107.200%. The results demonstrate the satisfactory recoveries of the GFET toward β-gal. We believe that our GFET sensing platform has great potential for the identification and analysis of environmental water sources in the future.

## 4. Conclusions

In summary, we have demonstrated that the here-developed functionalized GFET biosensor can achieve ultrasensitive and label-free detection of β-gal produced by *E. coli*. The universal graphene sensing interface modified with heat-denatured casein was developed, which has been used to connect different biological capture probes and prevent non-specific adsorption of interfering substances in the water sample. The sensing detection of β-gal was performed by exposing the sensor to various water samples to monitor environmental water sources and was able to determine the presence of E. coli in less than 10 min. Furthermore, the GFET provides a platform which is amenable to mass production and has a low manufacturing cost while saving time; this device can quantitatively and specifically detect low-abundance β-gal with a good linear range from 1 fg·mL^−1^ to 100 ng·mL^−1^, with a detection limit of 0.187 fg·mL^−1^ (1.61 aM). It is also of note that the surface of GFET, functionalized by the high selectivity of the anti-β-gal antibody, can specifically recognize β-gal. The proposed GFET biosensor has excellent sensing performance, making it an ideal tool for monitoring the presence of *E. coli* in environmental water sources. There is substantial potential here for commercial applications in future development of POCT devices. In the future, it may be possible to develop a portable testing platform, combined with the chip developed by us, which will enable environmental regulators to obtain test results more quickly, provide results efficiently and formulate effective response plans upon detection of polluted water sources. The platform may effectively eliminate the need to send samples to a central laboratories for testing, greatly improving regulatory authorities’ chances of obtaining accurate test results in remote areas.

## Figures and Tables

**Table 1:** Analysis of β-gal in spiked river water samples by the GFET biosensor.

Sample	Found	Added (ng·mL^−1^)	Recovered (ng·mL^−1^)	Recovery
River 1	Not detected	1	1.072 ± 0.317	107.200%
River 2	Not detected	10	9.271 ± 0.281	92.710%
River 3	Not detected	100	98.729 ± 0.836	98.729%

## Data Availability

The data presented in this study are available on request from the corresponding author.
